# Acute Pancreatitis in a Patient With Recent History of SARS-CoV-2 Infection

**DOI:** 10.7759/cureus.29032

**Published:** 2022-09-11

**Authors:** Victoria Reick-Mitrisin, Kashif Mukhtar, Zarak H Khan

**Affiliations:** 1 Medical Education, A.T. Still University Kirksville College of Osteopathic Medicine (ATSU-KCOM), Kirksville, USA; 2 Internal Medicine, St. Mary Mercy Hospital, Livonia, USA; 3 Gastroenterology, East Carolina University, Greenville, USA

**Keywords:** covid-19 complications, acute pancreatic injury, acute pancreatitis, covid-19, sars-cov-2

## Abstract

Acute pancreatitis (AP) is caused by inflammation of the exocrine pancreas. It is often due to loss of compartmentalization and subsequent activation of pancreatic enzymes prior to leaving the pancreatic duct. AP caused by viral infections is commonly referenced in the literature. The association of AP with SARS-CoV-2 has been reported in the past several months in both retrospective cohort studies and case reports. However, there is currently limited evidence regarding the incidence of AP in the setting of SARS-CoV-2. We present a unique case of AP as an early complication in a patient three days after hospitalization for SARS-CoV-2. It is imperative to consider AP in the differential diagnoses of patients with a recent history of SARS-CoV-2 infection presenting with acute abdominal pain.

## Introduction

Acute pancreatitis (AP) occurs due to an increase in trypsin or a decrease in trypsinogen activation [1]. This can be due to obstructive or inflammatory mechanisms, such as biliary tract obstruction, or a recent inflammatory process, such as a viral infection. The mechanism involves trypsin reflux from the pancreatic duct and subsequent activation, leading to the digestion of pancreatic parenchyma [2]. The most common cause of AP is bile duct obstruction (38% of cases), followed by alcohol abuse (36% of cases) in the US population [2]. Other causes include pancreas divisum, hypertriglyceridemia, recent endoscopic retrograde cholangiopancreatography (ERCP), malignancies, drugs, or inflammatory processes such as viral infections [2].

Virus-induced AP has been extensively studied previously. Viral hepatitis accounts for most of the cases of virus-induced AP. Other viruses that have been previously shown to cause AP include cytomegalovirus, varicella-zoster virus, coxsackie virus, echoviruses, measles, mumps, HIV infection, HSV, EBV, adenovirus, influenza, and H1N1 [3]. Emerging literature has also shown reports of AP in patients with SARS-CoV-2 infection. We present a case of pancreatitis in a patient who was recently admitted for SARS-CoV-2 pneumonia. The patient presented with AP three days after discharge from the hospital. This case has previously been presented at the World Congress of Gastroenterology organized by the American College of Gastroenterology in October 2021 [4].

## Case presentation

A 71-year-old male with a past medical history of type 2 diabetes mellitus, end-stage renal disease on hemodialysis, status-post renal transplantation with subsequent failure, paroxysmal atrial fibrillation, history of colon diverticulitis with perforation status-post colostomy and multiple other co-morbidities who presented to the emergency room with acute onset epigastric abdominal pain. His abdominal pain was sharp and non-radiating with no associated nausea or vomiting. He was discharged three days prior after a nine-day admission for SARS-CoV-2 pneumonia. During his previous admission, he received a five-day course of remdesivir (completed on day 6 of admission) and a dose of tocilizumab (on day 4 of admission). He was also started on a 10-day course of dexamethasone which he was instructed to complete at home upon discharge (two doses left at discharge) (Table 1). After a significant improvement in his condition, he was discharged home on 3 liters of oxygen through a nasal cannula. The patient denied any history of smoking, drinking, or illicit drugs use. His home medications included allopurinol, pantoprazole, apixaban, atorvastatin, vitamin B complex, vitamin C, folic acid, carvedilol, digoxin, ibuprofen, insulin, levothyroxine, prednisone, tamsulosin, calcium acetate, and zolpidem all of which he was taking for several years (Table 2).

**Table 1:** Medications at discharge.

Medications	Instructions
Dexamethasone (DECADRON) 6 mg tablet	1 tablet by mouth 1 (one) time a day for 2 days
AllopurinoL (ZYLOPRIM) 100 mg tablet	1 tablet by mouth 1 (one) time a day
Apixaban (Eliquis) 2.5 mg tablet	1 tablet by mouth 2 (two) times a day
Atorvastatin (LIPITOR) 10 mg tablet	1 tablet by mouth 1 (one) time a day
B complex-vitamin C-folic acid (NEPHROCAPS) 1 mg capsule	1 capsule by mouth 1 (one) time a day
Betamethasone dipropionate (DIPROLENE) 0.05 % cream	As needed three times a day
Calcium acetate, phosphate binder, (PHOSLO) 667 mg capsule	2 capsules by mouth 3 (three) times a day with meals. 2 capsules daily with snack
Digoxin (LANOXIN) 125 mcg (0.125 mg) tablet	1 tablet by mouth 3 (three) times a week: Tuesday, Thursday, Saturday
Doxercalciferol (HECTOROL) 1 mcg capsule	1 capsule by mouth 1 (one) time a day
Hydrocortisone 2.5 % cream	Apply to affected area 1 (one) time a day
Insulin NPH-insulin regular (HumuLIN, 70/30,) 100 unit/mL (70-30) injection	Inject under skin 2 (two) times a day. 15 units in the morning, and 10 units in the evening and as needed
Levothyroxine sodium (TIROSINT) 125 mcg capsule	1 capsule by mouth 1 (one) time a day
Midodrine (PROAMATINE) 10 mg tablet	1 tablet by mouth 2 (two) times a day
Tamsulosin HCl (TAMSULOSIN ORAL)	1 tablet by mouth 1 (one) time a day
Zolpidem CR (AMBIEN CR) 12.5 mg CR tablet	1 tablet by mouth at night if needed for sleep. Do not crush, chew, or split

**Table 2:** Medication summary during the hospital stay.

Medication	Frequency	Route	Start date	End date
Acetaminophen (TYLENOL) tablet 500 mg	Every 6 hours as needed	PO	04/16/21	04/24/21
AllopurinoL (ZYLOPRIM) tablet 100 mg	Daily	PO	04/16/21	04/24/21
Apixaban (ELIQUIS) tablet 2.5 mg	2 times daily	PO	04/16/21	04/24/21
Ascorbic acid (VITAMIN C) tablet 500 mg	Daily	PO	04/16/21	04/24/21
Atorvastatin (LIPITOR) tablet 10 mg	Nightly	PO	04/16/21	04/24/21
B complex-vitamin C-folic acid (NEPHROCAPS) capsule 1 capsule	Daily	PO	04/16/21	04/24/21
Dexamethasone (DECADRON) tablet 6 mg	Daily	PO	04/16/21	04/24/21
Digoxin (LANOXIN) tablet 125 mcg	Once per day on Tue Thu Sat	PO	04/16/21	04/24/21
Insulin lispro (HumaLOG) injection 3-18 Units	Three times daily with meals	IM	04/16/21	04/24/21
Levothyroxine tablet 125 mcg	Every morning	PO	04/16/21	04/24/21
Midodrine (PROAMATINE) tablet 5 mg	Two times daily	PO	04/16/21	04/24/21
Nystatin (MYCOSTATIN) 100,000 unit/mL suspension 500,000 Units	4 times daily	PO, swish and swallow	04/16/21	04/24/21
Remdesivir (VEKLURY) 100 mg in sodium chloride 0.9 % 270 mL IVPB	Every 24 hours	IV	04/17/21	04/21/21
Tocilizumab (ACTEMRA) 600 mg in sodium chloride 0.9 % 100 mL IVPB	Once	IV	04/19/21	04/19/21
Dextromethorphan-guaiFENesin (ROBITUSSIN-DM) 10-100 mg/5 mL syrup 10 mL	Every 6 hours	PO	04/16/21	04/24/21
Melatonin tablet 3 mg	Nightly	PO	04/21/21	04/23/21
Zinc sulfate (ZINCATE) capsule 220 mg	Daily	PO	04/16/21	04/24/21

On arrival, his vital signs were remarkable for tachycardia. A physical exam was significant for an ill-appearing man with a non-distended abdomen and tenderness in the epigastric region with voluntary guarding. The patient's laboratory workup was significant for an elevated lipase of 5282 U/L (table 3).

**Table 3:** Patient's lab work during the hospital stay.

Investigations	Results	Reference Range & Units
Sodium	137	136 - 145 mmol/L
Potassium	3.9	3.5 - 5.3 mmol/L
Chloride	96	98 - 107 mmol/L
Bicarbonate	25	21 - 31 mmol/L
Glucose	208	70 - 100 mg/dL
Blood Urea Nitrogen (BUN)	72.8	7.0 - 25.0 mg/dL
Creatinine	5.81	0.60 - 1.20 mg/dL
GFR	10	59 - 180 mL/min
Calcium	8.2	8.6 - 10.3 mg/dL
Protein Total	6.4	6.4 - 8.2 gm/dL
Albumin	3.4	3.5 - 5.7 gm/dL
Bilirubin Total	0.7	0.1 - 1.0 mg/dL
Aspartate Aminotransferase	36	13 - 39 U/L
Alanine Aminotransferase	20	7 - 52 U/L
Lipase	5,282	11 - 82 U/L
Lactate	1.9	0.4 - 2.0 mmol/L
White Blood Cells	10.9	3.6 - 11.1 K/UL
RBC Count	2.67	4.30 - 5.90 M/UL
Hemoglobin	9.2	12.9 - 18.0 dm/dL
Hematocrit	29.6	37.6 - 52.0 %
MCV	111	82 - 102 FL
MCHC	31.1	30.0 - 36.0 gm/dL
Platelet Count	167	140 - 440 K/UL
RDW	15.2	12.0 - 16.0 %
CRP	1.9	< 1.0 mg/dL
ESR	49	< 20 mm/h
Triglycerides	457	< 150 mg/dL
HbA1c	6.3	< 6.0 %
TSH	0.62	0.4 – 4.0 IU/L
Free T4	1.07	0.7 – 1.8 ng/dL
IgG4	9	3 – 201 mg/dL

**Figure 1:**
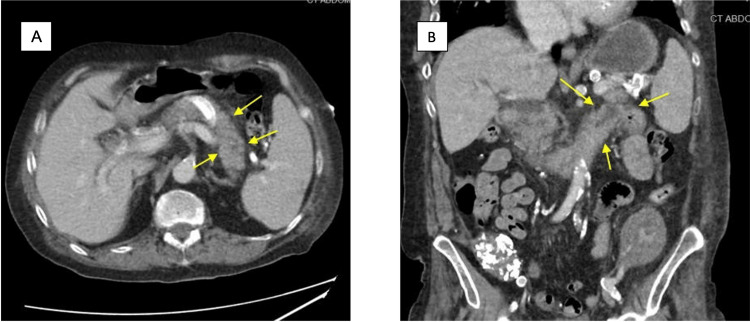
CT scan images of transverse (A) and coronal (B) sections showing edematous body and tail of pancreas (yellow arrows).

An ultrasound was not able to visualize the pancreas due to bowel gas. The gallbladder was surgically absent on the ultrasound. CT scan showed fat stranding adjacent to the pancreas, mild splenomegaly, and bilateral ground glass opacities in the lungs, as well as consolidations in the mid and lower lungs concerning atypical pneumonia (Figures 1A, 1B). He met all three criteria as per the revised Atlanta classification (acute epigastric pain, elevated lipase greater than three times the normal limit, and characteristic imaging findings) for AP. Based on his CT scan findings, he met the criteria for Balthazar grade C (CT severity index: 2), which was consistent with mild AP.

The patient was managed conservatively with intravenous fluids and pain medications. During his hospitalization, the patient developed odynophagia, which improved with pantoprazole and lozenges. A repeat CT scan of the patient’s abdomen and pelvis was performed due to concern for hemorrhagic conversion of pancreatitis due to worsening anemia. It was negative for conversion and showed resolution of pancreatitis. The patient was discharged with a home healthcare service and informed to follow up with his primary care physician two to three days after discharge. He was doing well on his follow-up visit.

## Discussion

There is currently limited information regarding SARS-CoV-2-associated pancreatitis, however recent literature shows an increasing incidence. Several case reports have recently been published that have demonstrated this association [5-7]. Wang et al. presented a case series of 52 cases in which 17% of patients with severe SARS-CoV-2 developed pancreatic injury evident by elevated amylase or lipase levels. 7% of these patients had evidence of AP in imaging studies [8]. One retrospective study that utilized a public database in Wuhan China also reported the same percentage of 17% pancreatic damage in their patients with severe infection [9]. It is worth noting that some of these patients have been on multiple medications including corticosteroids and non-steroidal anti-inflammatory drugs (NSAIDs) which are independent risk factors for pancreatitis. It is also worth noting that the AP is very rarely the presenting feature but instead develops during the course of the disease. In most of the previous cases, the acute pancreatic injury was seen during the course of the SARS-CoV-2 infection while in our case it was seen as an early complication. AP as an early complication of SARS-CoV-2 has not been extensively studied.

The SARS-CoV-2 virus uses angiotensin-converting enzyme 2 (ACE2) and transmembrane serine protease 2 (TMPRSS2) to enter cells. These receptors are present in abundance on gastrointestinal epithelial cells, as well as pancreatic ductal and acinar cells [9,10]. This association makes the likelihood that SARS-CoV-2 can induce pancreatitis more plausible due to the shared surface proteins and increased ability to enter enteric and pancreatic cells. Studies have shown that patients who get AP have increased expression of ACE2 receptors in their pancreatic cells compared to non-infected patients [9]. However, this is a suggested association and further studies are warranted to investigate this association and other associations that can contribute to pancreatic injury in patients with SARS-CoV-2 infection.

Due to our patient’s complex clinical course and past medical history, he had several risk factors that could have also contributed to his AP including the use of various medications. He received tocilizumab, which may be associated with AP [11]. However, only a dearth of evidence exists in the literature that looks at this co-relation. He was also taking prednisone and atorvastatin which have also been previously associated with pancreatitis [12-16]. Since drug-induced pancreatitis can occur at any time between hours and years, it is essentially impossible to rule out drug-induced pancreatitis [16]. Although further studies are required to investigate SARS-CoV-2-related AP, the clinical presentation for both SARS-CoV-2 and AP can be overlapping. Therefore, patients presenting with AP should always be investigated for SARS-CoV-2 infection and vice versa if patients with SARS-CoV-2 are presenting with signs and symptoms of AP like abdominal pain, nausea, vomiting, and fever. This will help identify both pathologies at an early stage and treatment can be started before complications have occurred.

## Conclusions

AP has been well-documented in the setting of viral illnesses. Emerging evidence suggests that SARS-CoV-2 is a possible trigger for inflammatory processes that place patients at increased risk for AP in the future. In order to continue to determine if SARS-CoV-2 has an increased association with AP, we would require additional understanding of the clinical course and etiology behind SARS-CoV-2-induced pancreatitis as well as the risk factors and clinical course associated with increased risk of pancreatitis post-infection. Additionally, various medications used for SARS-CoV-2 should be further investigated to determine a correlation between their use and increased or decreased incidence of post-infectious AP. This should include remdesivir, tocilizumab and dexamethasone, as were used for this patient during their hospitalization for SARS-CoV-2, in addition to his history of chronic use of oral steroids. AP should be considered in the differential of abdominal pain in patients with active or recent SARS-CoV-2 infection.
